# Modulatory role of androgenic and estrogenic neurosteroids in determining the direction of synaptic plasticity in the CA1 hippocampal region of male rats

**DOI:** 10.1002/phy2.185

**Published:** 2013-12-08

**Authors:** Vito Enrico Pettorossi, Michela Di Mauro, Mariangela Scarduzio, Roberto Panichi, Alessandro Tozzi, Paolo Calabresi, Silvarosa Grassi

**Affiliations:** 1 Dipartimento di Medicina Interna, Sezione di Fisiologia Umana, Università di Perugia, Polo Unico Sant'Andrea delle Fratte, Via Gambuli, Perugia, 106156, Italy; 2 Clinica Neurologica, Ospedale S. Maria della Misericordia, Università di Perugia, Perugia, 06156, Italy; 3 Fondazione Santa Lucia, I.R.C.C.S, Roma, 00143, Italy

**Keywords:** Androgen receptors, estrogen receptors, hippocampus, long‐term depression, long‐term potentiation, sex neurosteroids

## Abstract

Estrogenic and androgenic neurosteroids can rapidly modulate synaptic plasticity in the brain through interaction with membrane receptors for estrogens (ERs) and androgens (ARs). We used electrophysiological recordings in slices of young and adolescent male rats to explore the influence of sex neurosteroids on synaptic plasticity in the CA1 hippocampal region, by blocking ARs or ERs during induction of long‐term depression (LTD) and depotentiation (DP) by low‐frequency stimulation (LFS) and long‐term potentiation (LTP) by high‐frequency stimulation (HFS). We found that LTD and DP depend on ARs, while LTP on ERs in both age groups. Accordingly, the AR blocker flutamide affected induction of LTD reverting it into LTP, and prevented DP, while having no effect on HFS‐dependent LTP. Conversely, ER blockade with ICI 182,780 (ICI) markedly reduced LTP, but did not influence LTD and DP. However, the receptor blockade did not affect the maintenance of either LTD or LTP. Moreover, we found that similar to LTP and LTD induced in control condition, the LTP unveiled by flutamide during LFS and residual LTP induced by HFS under ICI depended on *N*‐methyl‐d aspartate receptor (NMDAR) activation. Furthermore, as the synaptic paired‐pulse facilitation (PPF) was not affected by either AR or ER blockade, we suggest that sex neurosteroids act primarily at a postsynaptic level. This study demonstrates for the first time the crucial role of estrogenic and androgenic neurosteroids in determining the sign of hippocampal synaptic plasticity in male rat and the activity‐dependent recruitment of androgenic and estrogenic pathways leading to LTD and LTP, respectively.

## Abstract (precis)

This study shows a crucial and opposite role of estrogenic and androgenic neurosteroids in guiding the direction of synaptic plasticity in the hippocampus CA1 region of male rat, through activation of their specific receptors. In fact, by using selective antagonists for estrogen receptors (ICI 182,730) or androgen receptors (flutamide), we show that long‐term potentiation (LTP) induced by high‐frequency stimulation (HFS) depends on estrogenic signals, while long‐term depression (LTD) and depotentiation induced by low‐frequency stimulation (LFS) require activation of androgenic pathway. We suggest that different stimulation frequencies may lead to LTD or LTP depending on activation of specific neurosteroid pathway.

## Introduction

17*β*‐estradiol (E2), testosterone (T), and 5*α*‐dihydrotestosterone (DHT) are neuroactive steroids, which rapidly modulate synaptic transmission and plasticity in the brain (McEwen 2002; Isgor and Sengelaub 2003; MacLusky et al. 2006) through fast non‐genomic mechanisms mediated by direct interaction with membrane receptors for E2 (estrogens [ERs]: *α* and *β*) and androgens (ARs) (Tabori et al. 2005; Pedram et al. 2006; Foradori et al. 2008; Morisette et al. 2008; Raz et al. 2008; Levin 2009). In addition to circulating steroids of gonadal origin, sex steroids are synthesized in the nervous systems of both genders from cholesterol (Baulieu 1997; Compagnone and Mellon 2000) through pregnenolone and dehydroepiandrosterone generating T that is then converted into E2 and DHT by P450‐aromatase and 5*α*‐reductase enzymes, respectively (Selmanoff et al. 1997; Kimoto et al. 2001; Hojo et al. 2004; Mukai et al. 2006; Hojo et al. 2008). Neural E2 and DHT are believed to play an important role as region‐specific modulatory neurosteroids as they reach relatively high concentrations in the brain (Kimoto et al. 2001; Hojo et al. 2004; Mukai et al. 2006; Hojo et al. 2008, 2009). For instance, the concentrations of E2 and DHT determined in hippocampus of male rat (8.4 and 6.6 nmol/L, respectively, Hojo et al. 2008, 2009) are significantly higher than those at circulating level, and E2 or DHT, exogenously used at similar concentrations, are able to influence the synaptic response and plasticity. In fact, it has been shown that E2 increases the neurotransmission mediated by the NR2B containing *N*‐methyl‐d aspartate receptors (NMDARs) at the glutamatergic synaptic level and the magnitude of long‐term potentiation (LTP) at hippocampus CA3‐CA1 synapses (Woolley et al. 1997; Wong and Moss 1992; Foy et al. 1999; Foy 2001; Smith and McMahon 2005, 2006; Smith et al. 2009), while T and DHT tend to reduce it (Harley et al. 2000; Hebbard et al. 2003), suggesting an opposite role of estrogenic and androgenic neurosteroids on synaptic plasticity. A direct evidence for an involvement of locally synthesized E2 in the activity‐dependent synaptic plasticity has been recently provided by Grassi et al. (2009), who demonstrated, for the first time, that induction of LTP by high‐frequency stimulation (HFS) in the vestibular nuclei was prevented by blockade of either P450‐aromatase or ERs, a result also confirmed in the hippocampus of adult male rat (Grassi et al. 2011; Tanaka and Sokabe 2012).

Conversely, whether local synthesis of androgenic steroids (T and DHT) is directly implicated in synaptic plasticity driven by neuronal activity is still unknown. This possibility is suggested by the effects of exogenously administered T in vestibular slices that can induce LTP or long‐term depression (LTD), depending on its respective conversion into E2 or DHT (Grassi et al. 2010a). In addition, the recent findings that vestibular neurons coexpress ARs and ERs and develop LTP, followed by LTD, when E2 and DHT are successively administered (Grassi et al. 2013), suggest that the direction of synaptic long‐term effects in the same neuron may be established by the activity‐dependent release of androgenic or estrogenic neurosteroids. Therefore, we hypothesize that different patterns of synaptic activation may specifically guide the synthesis of E2 or DHT and induce LTP or LTD. We already know that both LTP induced by HFS in vestibular and hippocampus neurons require the neural synthesis of E2 and activation of ERs for their full expression (Grassi et al. 2009, 2011; Tanaka and Sokabe 2012), but we have no evidence for the involvement of sex neurosteroids, and in particular ARs, in the LTD induced by low‐frequency stimulation (LFS).

Therefore, we investigated in the hippocampal CA1 region, the role of ARs and ERs in the activity‐dependent long‐term synaptic plasticity. In this region, HFS of Schaffer collateral fibers induces LTP, while LFS causes LTD and depotentiation (DP) of prior evoked LTP (Staubli and Lynch 1990; Dudek and Bear 1992, 1993; Bear and Malenka 1994; Staubli and Ji 1996). As hippocampus is equipped with the full enzymatic machinery to produce T, and therefore E2 and DHT de novo (Hojo et al. 2004; Mukai et al. 2006; Hojo et al. 2008, 2009), and it also expresses widely ERs and ARs (Kerr et al. 1995; Kalita et al. 2005), we investigated in hippocampal slices of male rats, the effect of AR and ER blockade during stimulation protocols inducing LTD, LTP, and DP.

## Material and Methods

### Ethical approval

All procedures on animals were performed in strict accordance with protocols approved by the Ethics Committees of the University of Perugia, with the guidelines of the Italian Ministry of Health, national laws on animal research (Legislative Decree 116/92) and the European Communities Council directive on animal research (N. 86/609/ECC). All efforts were made to minimize the number of animals used and their suffering.

### Slice preparation

The study was conducted in 276 hippocampal slices prepared from 125 male Wistar rats (Harlan, Italy) divided into two age groups: young animals at postnatal day <P30 (P14‐21, 217 slices, 91 animals) and adolescent ones at P50 (61 slices, 34 animals). We used animals of different age as induction of either LTD or DP is known to be age‐dependent. In fact, LTD is more prominent in relatively young animals (<P30) and DP in older ones (>P30), while LTP is easily induced in both age groups (Dudek and Bear 1993; Staubli and Ji 1996). In addition, we only used male rats to avoid any possible influence of cyclic, systemic estrogenic fluctuation on the induction of synaptic plasticity (Warren et al. 1995; Good et al. 1999). Animals were decapitated under anesthesia with halothane and the brain removed and immersed for 2–3 min in ice‐cold artificial cerebrospinal fluid (ACSF) containing (in mmol/L): 126 NaCl, 2.5 KCl, 1.2 MgCl_2_, 1.2 NaH_2_PO_4_, 2.4 CaCl_2_, 10 glucose, and 25 NaHCO_3_, continuously bubbled with 95% O_2_ and 5% CO_2_, pH 7.4. After hippocampus extraction, 400‐*μ*m‐thick transverse slices were cut in ice‐cold ACSF with a vibratome (Series 1000 plus starter CE; Vibratome, St. Louis, MO) and allowed to recover in oxygenated ACSF at room temperature for 2 h before experimental recordings.

### Electrophysiology

For each animal, we used 2–3 slices. A slice was transferred into the recording chamber and submerged with ACSF at a constant rate of 2 mL/min at room temperature. Under visual control, a bipolar platinum‐iridium stimulating electrode was placed into the Schaffer collateral fibers, and the recording electrode (borosilicate glass capillaries, GC150F‐10; Harvard Apparatus, Holliston, MA) filled with 2mol/L NaCl (resistance, 10–15 MΩ) was inserted into CA1 region. Electrical pulses (duration: 70 *μ*sec and intensity: 20–50 *μ*A) were delivered at a frequency of 0.06 Hz as test stimulation. This stimulation evoked field excitatory postsynaptic potential (fEPSP) that was 50–70% of maximum slope. fEPSP was filtered at 3 kHz, digitized at 10 kHz, and stored on PC equipped with a data acquisition card (at‐MIO‐16E‐2; National Instruments, Austin, TX). An Axoclamp 2B amplifier (Molecular Devices, Sunnyvale, CA) was used for recordings.

In some preliminary recordings we verified that test stimulation prolonged for 40 min never elicited long‐term effects. Long‐term changes of synaptic plasticity were induced by delivering, after stable baseline recording for 10 min, LFS (900 pulses at 1 Hz) for LTD or DP, and HFS (100 pulses at 100 Hz: four bursts separated by 5 sec) for LTP. In some experiments, paired‐pulse facilitation (PPF) of fEPSP was evoked with paired‐pulse stimulation (PPS) protocol at 50 msec interstimulus and 50% of the maximal stimulus intensity.

### Drugs

We used the selective antagonists for ARs, flutamide (100 nmol/L); ERs (*α* and *β*), ICI 182,780 (ICI, 100 nmol/L), and NMDARs, D‐(−)‐2‐amino‐5‐phosphonopentanoic acid (AP‐5, 100 *μ*mol/L), which were purchased from Sigma‐Aldrich (St Louis, MO). Flutamide is commonly used to block ARs but it can also influence GABAergic transmission, because of its similarity with benzodiazepines (Ahmadiani et al. 2003). However, this effect can be excluded as we used a concentration much lower than that reported to have anticonvulsant effects (Ahmadiani et al. 2003). Concerning ICI, it is a well‐known antagonist of nuclear ERs, however, it also acts as a membrane ER antagonist mediating rapid estrogenic effects (Wade et al. 2001; Micevych and Mermelstein 2008). Thus, we used ICI to block ER*α* and ER*β* localized at the cell membrane (Kalita et al. 2005; Levin 2009).

Stock solutions of flutamide (10 mmol/L) and ICI (1 mmol/L) were dissolved in dimethyl sulfoxide (DMSO) and that of AP‐5 (10 mmol/L) in distilled water. Drugs were diluted to working concentration in oxygenated ACSF and perfused at a rate of 2 mL/min. The final concentration of DMSO was 0.001%. Total replacement of the medium in the chamber occurred within 1 min. In the experiments in which induction of LTP and LTD was analyzed under ER and AR blockers, the drugs were applied for all the recording period commencing from 10 min before the start of recording and about 20 min before LFS or HFS application.

### Data analysis and statistical evaluation

To characterize the drug effects on the baseline fEPSP and on induction of the long‐term effects by LFS or HFS, the stimulation was applied every 15 sec. We measured the initial slope of fEPSP using linear regression of the first 0.8 msec succeeding the presynaptic fiber volley and used the average response recorded during a stable period (10 min) at the beginning of the experiment as the baseline. The averaged fEPSP calculated every minute was expressed as a percentage of the baseline fEPSP value and used for data presentation. In each experiment, we statistically verified (Student's paired *t*‐test) the occurrence of LTP and LTD by comparing the fEPSP slope at 40 min after stimulation with the baseline. The Student's paired *t*‐test was also used for evaluating the influence of drug application on the maintenance of long‐term effects, and for verifying the induction of DP. Moreover, to compare the effects observed in different experimental conditions, we used the one‐way analysis of variance (ANOVA) and the Tukey's post hoc test. In the experiments in which PPF was elicited, we calculated the paired‐pulse ratio (PPR) before and after drug application by dividing the mean slope of the second fEPSP by that of the first one. Student's paired *t*‐test was used to analyze changes in PPF.

The level of significance was set at *P* < 0.05 for Student's *t*‐test, ANOVA, and post hoc comparison. Statistical analyses were performed with Statistica (StatSoft, Tulsa, OK, USA).

Values given in the text are mean ± SE, *n* representing the slice number.

### Preliminary experiments

To exclude an influence of drug vehicle (0.001% DMSO) on the induction of LTD by LFS and LTP by HFS, we compared the occurrence and amplitude of these phenomena in slices of young animals during the bath perfusion with ACSF alone or ACSF plus DMSO. LTD and LTP were always induced in both conditions (for each condition: LFS, *n* = 5, three animals; HFS, *n* = 5, three animals) and their amplitude was not different (ANOVA, LTD: *F*_(1,28)_ = 0.5; *P* = 0.49; LTP: *F*_(1,25)_ = 0.57; *P* = 0.45).

We also analyzed the influence of the AR and ER antagonists on the baseline. No significant modification was observed in the presence of flutamide, ICI, or flutamide plus ICI (*n* = 6, three animals for each condition).

## Results

### Effect of AR and ER blockade on the induction of LTD in young rats

We analyzed the possible involvement of ARs and E2 on the induction of LTD by LFS in young (P14‐P21) rats, an age in which LTD results to be more prominent compared with the adult (Dudek and Bear 1993). The LFS protocol was, therefore, applied in control slices and in the presence of flutamide or ICI, the selective antagonists for the ARs and ERs, respectively.

**Figure 1.**
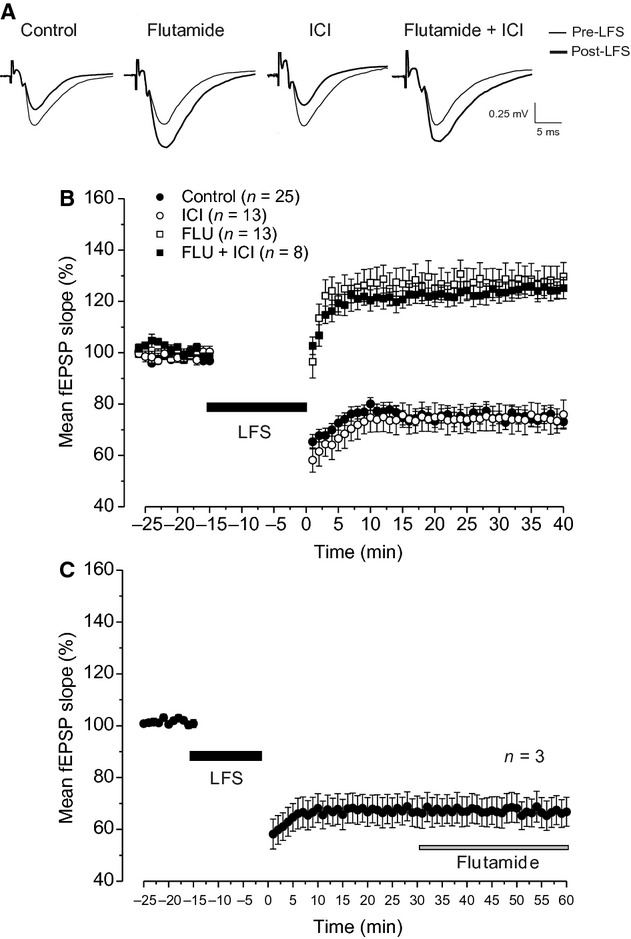
Effects of AR and ER blockade on LTD induced by LFS in young rats. (A) Averaged traces (*n* = 20) of fEPSPs recorded before (thin traces, pre‐LFS) and 40 min after LFS (thick traces, post‐LFS) in control condition and under flutamide, ICI, and flutamide plus ICI. (B and C) Time courses of the effects of LFS on the fEPSP. In this figure and in the ones that follow, points represent the mean ± SE (*n* = number of slices) of the fEPSP slope evaluated within 1‐min interval and expressed as a percentage of baseline. (B) The effects of LFS in control condition (filled circles) and in the presence of flutamide (open squares), ICI (open circles), or flutamide plus ICI (filled squares). (C) The effect of flutamide on the maintenance of LFS‐LTD. The black bars show the LFS delivering time and the gray bar the flutamide infusion time. AR, androgenic receptor; ER, estrogen receptor; LTD, long‐term depression; LFS, low‐frequency stimulation; ICI, ICI 182,780; fEPSP, field excitatory postsynaptic potential.

In control condition, the application of the LFS protocol induced LTD (76.7 ± 2.3%, *n* = 25, 12 animals) while in the presence of flutamide, the same protocol evoked LTP (133.3 ± 5.3%, *n* = 13, six animals, Fig. 1A and B) that was significantly lower than that induced by HFS under control condition (154.6 ± 3.6% see below, ANOVA: *F*_(1,33)_ = 11.5; *P* = 0.0017, Fig. 1B). Conversely, LTD was not altered by ICI (74.9 ± 5.6%, *n* = 13, seven animals, ANOVA: *F*_(1,36)_ = 0.3; *P* = 0.58, Fig. 1A and B) and LFS applied in the presence of flutamide plus ICI induced LTP that was similar to that obtained in the presence of flutamide alone (123.1 ± 4%, *n* =**8, four animals, Fig. 1A and B, ANOVA: *F*_(1,20)_ = 1.96; *P* = 0.17) suggesting that E2 is not involved in LTP induced by LFS under AR blockade.

Moreover, to address whether the AR blockade could affect the maintenance of LTD, flutamide was applied 30 min after the induction of LTD (*n *=**3, two animals). We found that LTD was not changed by flutamide. In fact, the values of LTD before (65.3 ± 6%) and 30 min after drug application (66.7 ± 5.6%) were not significantly different (Student's *t*‐test, *P *=**0.14, Fig. 1C).

### Effect of AR and ER blockade on the induction of LTP in young rats

**Figure 2.**
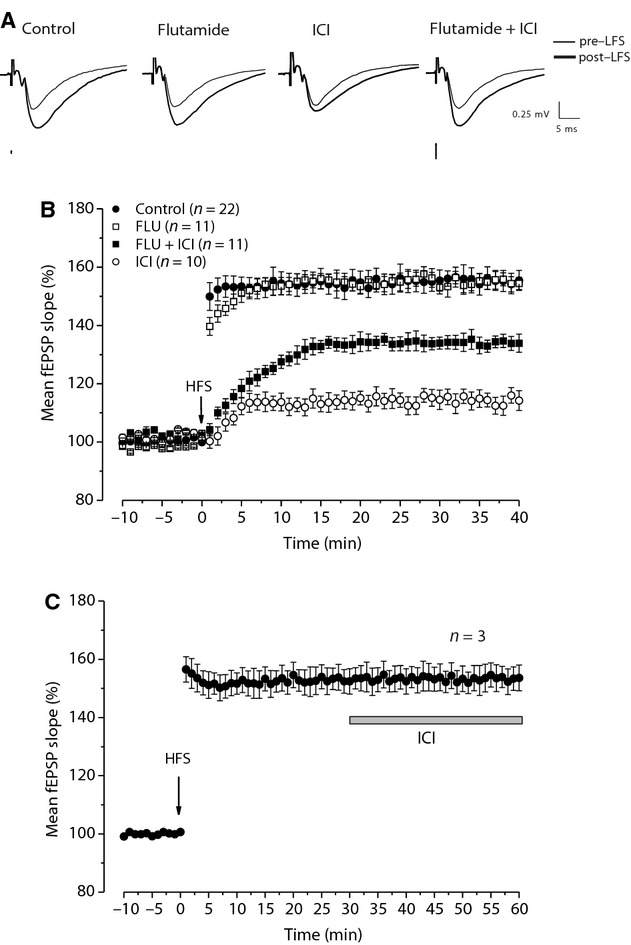
Effects of AR and ER blockade on LTP induced by HFS in young rats. (A) Averaged traces (*n* = 20) of fEPSPs recorded before (thin traces, pre‐HFS) and 40 min after HFS (thick traces, post‐HFS) in control condition and under flutamide, ICI, and flutamide plus ICI. (B) Time courses of the HFS effects in control condition (filled circles) and in the presence of flutamide (open squares), ICI (open circles), and flutamide plus ICI (filled squares). (C) The effect of ICI on the maintenance of HFS‐LTP. The arrows indicate the HFS delivering time and the gray bar the ICI infusion time. AR, androgenic receptor; ER, estrogen receptor; LTP, long‐term potentiation; HFS, high‐frequency stimulation; ICI, ICI 182,780; fEPSP, field excitatory postsynaptic potential.

In order to study the possibility that ARs and E2 could also influence LTP in the hippocampal CA1 region of young rats through interaction with ERs and ARs, we first induced this form of synaptic plasticity by applying the HFS‐stimulating protocol in control condition and in the presence of the antagonists of ERs (ICI) or/and ARs (flutamide). LTP measured in the presence of ICI (114.3 ± 2.3%, *n *=**10, six animals) was significantly smaller than that induced in control condition (154.6 ± 3.6%, *n *=**22, eight animals, Fig. 2A and B), in full agreement with the observation obtained in adolescent rats in this study (see below) and in our previous study (Grassi et al. 2011). Moreover, blockade of ARs with flutamide did not affect the amplitude of LTP with respect to control (154 ± 2.5%, *n *=**11, seven animals, Fig. 2B). Conversely, LTP induced under combined blockade of ARs and ERs (flutamide plus ICI) was reduced in amplitude compared to control (132.6 ± 2.4%, *n *=**11, five animals), but it was significantly larger than that one obtained in the presence of ICI alone (ANOVA: *F*_(1,50)_ = 28.9, *P *=**0.00001; Tukey's post hoc test: ICI vs. control: *P *=**0.00016, flutamide vs. control: *P *=**0.99, flutamide + ICI vs. control: *P *=**0.00027, ICI vs. flutamide: *P *=**0.00016, flutamide + ICI vs. ICI: *P *= 0.006; Fig. 2A and B), suggesting a depressant effect of ARs when HFS was applied under blockade of ERs. Interestingly, LTP elicited by HFS in the presence of flutamide plus ICI was significantly larger than the LTP induced by LFS in the presence of flutamide plus ICI (ANOVA: *F*_(1,18)_ = 5.36; *P* = 0.032).

We also verified the possible effect of the ER blockade on the maintenance of LTP by applying ICI 30 min after its induction by HFS (*n *=**3, two animals). ICI did not significantly change the potentiation as its amplitude before ICI (154.3 ± 3.1%) was not significantly different from that measured 30 min after ICI (153.3 ± 4.1%, Student's *t*‐test, *P *=**0.22, Fig. 2C).

### Effect of AR and ER blockade on the induction of LTD and LTP in adolescent rats

**Figure 3.**
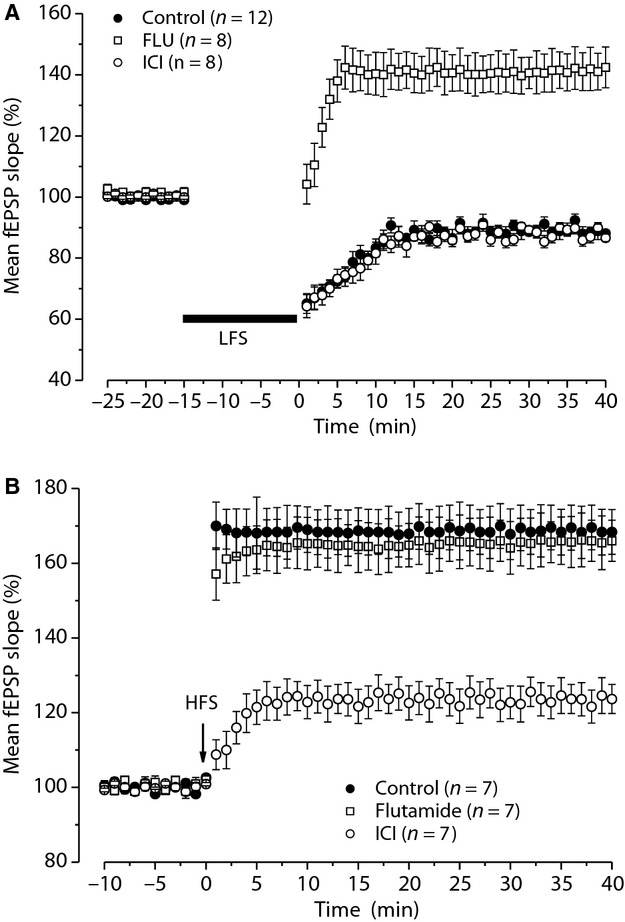
Effects of AR and ER blockade on induction of LTD and LTP in adolescent rats. (A and B) Time course of the LFS effects (A) and the HFS effects (B) in control condition (filled circles) and in the presence of flutamide (open squares) or ICI (open circles). AR, androgenic receptor; ER, estrogen receptor; LTD, long‐term depression; LFS, low‐frequency stimulation; LTP, long‐term potentiation; HFS, high‐frequency stimulation; ICI, ICI 182,780.

We verified whether AR and ER activation was also involved in the induction of long‐term synaptic plasticity in the adolescent (P50) rats. We first analyzed induction of LTD by LFS protocol that is a less prominent phenomenon in the adult compared to the young rat (Dudek and Bear 1993; Staubli and Ji 1996). Unlike rats at <P30, in which LFS always elicited LTD, in the adolescent rats LFS induced LTD in only 70% of the cases (12 of 17 slices, five animals). Moreover, the amplitude of this LTD (88.1 ± 1.1%) was significantly lower than that obtained in young rats (ANOVA: *F*_(1,35)_ = 7.3; *P *=**0.01, Fig. 3A). However, as observed in young rats, LFS in the presence of flutamide induced LTP (142.4 ± 6.6%, 8 of 11 slices, four animals) while, under ICI, it still evoked LTD (87.1 ± 1.25%, 8 of 11 slices, four animals) with an amplitude not significantly different from that observed under control condition (ANOVA: *F*_(1,18)_**= 0.34; *P *=**0.56, Fig. 3A).

We also analyzed the effects of flutamide and ICI on the induction of LTP by HFS. As observed in young rats, LTP induced in the presence of flutamide (166 ± 5.4%, *n *=**7, three animals) was not significantly different from that observed in the control condition (168.3 ± 6%, *n *=**7, three animals, ANOVA: *F*_(1,12)_ = 0.042; *P* = 0.8, Fig. 3B). In contrast, as shown in our previous study (Grassi et al. 2011), LTP induced in the presence of ICI (123.6 ± 3.9%, *n *=**7, three animals) was significantly lower than the control one (ANOVA: *F*_(1,12)_ = 36.45, *P *=**0.00005, Fig. 3B).

### DP of synaptic plasticity depends on AR activation

**Figure 4.**
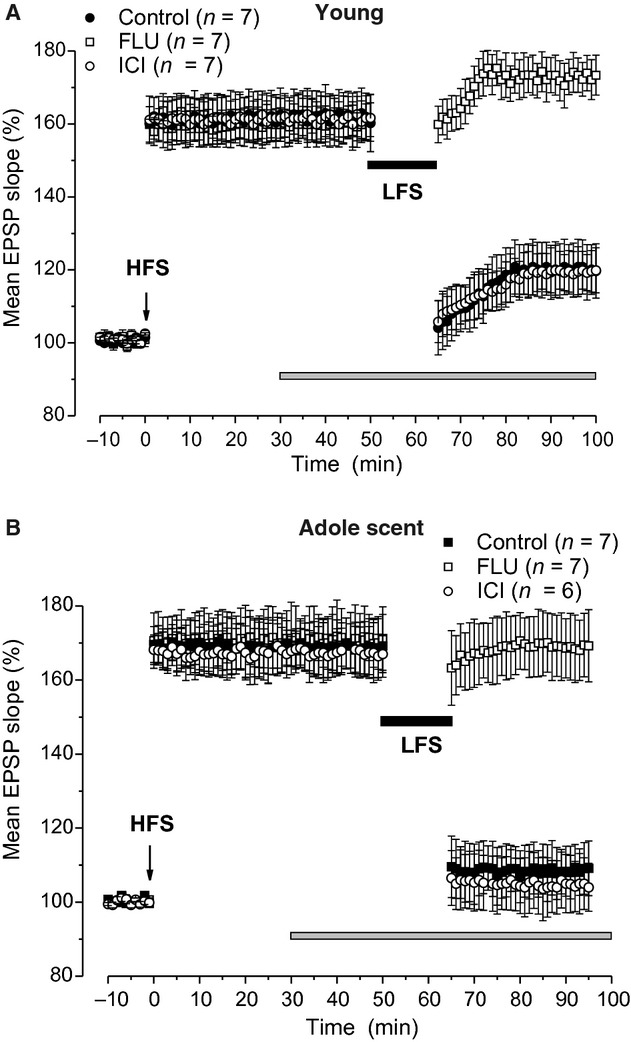
Effects of AR and ER blockade on DP induced by LFS in young and adolescent rats. (A and B) Time courses of the effects of LFS alone (control condition, filled circles), LFS + flutamide (open squares), and LFS + ICI (open circle) applied after induction of HFS‐LTP in young (A) and adolescent (B) rats. The gray bars indicate the drug infusion time. DP, depotentiation; LFS, low‐frequency stimulation; LTP, long‐term potentiation; HFS, high‐frequency stimulation; ICI, ICI 182,780.

Depotentiation of synaptic plasticity following the induction of LTP may be induced in the CA1 hippocampal area by LFS (Staubli and Lynch 1990). As it has been reported that DP is more evident in adult than in young animals (Dudek and Bear 1993), we examined this phenomenon and the effect of AR and ER blockade in both young and adolescent rats. In control condition, (*n *=**7, four animals, for each age group) LFS delivered 50 min after induction of LTP by HFS significantly reduced it in both age groups (Fig. 4A and B). In fact, fEPSP decreased from 163.5 ± 7% to 120.7 ± 7.6% (Student's *t*‐test, *P *=**0.0002) in young rats and from 168.3 ± 7.2% to 109.9 ± 7.1% (Student's *t*‐test, *P *=**0.001) in adolescent animals, values which were not significantly different (ANOVA: *F*_(1,12)_ = 1.09; *P *=**0.31). To assay the possible involvement of AR or ER activation in the induction of DP, LFS was delivered in the presence of flutamide or ICI. The drugs were administered starting from 10 min before LFS for more than 60 min. Interestingly, in the presence of flutamide (*n *=**7, four animals, for each age group), LFS was never able to induce DP in both animal groups (Fig. 4A and B). In fact, the fEPSP values measured before and after LFS were not significantly different (young: pre‐LFS 159.9 ± 5.2%, post‐LFS 173.6 ± 5.6%, Student's *t*‐test, *P *=**0.07; adolescent: pre‐LFS 170.7 ± 9.1%, post‐LFS 169.3 ± 9.8%, Student's *t*‐test, *P *=**0.6). In addition, no significant difference was observed between young and adolescent post‐LFS values (ANOVA: *F*_(1,12)_ = 0.14; *P *=**0.7).

Conversely, ICI (*n *=**7, four animals in both ages) did not affect DP as fEPSP was significantly reduced from 162 ± 6.2% to 119.8 ± 6.8% (Student's *t*‐test, *P *= 0.0005) in young rats and from 167.7 ± 6.3% to 104 ± 6.4% (Student's *t*‐test, *P *=**0.0006) in adolescent rats (Fig. 4A and B) values which were not significantly different (ANOVA: *F*_(1,12)_ = 3.62; *P *=**0.08) and also did not statistically differ from those obtained under control condition (ANOVA: young, *F*_(1,12)_ = 0.01; *P *=**0.91; adolescent: *F*_(1,12)_ = 0.029; *P *=**0.8).

### Synaptic plasticity induced by different stimulations and regulated by neurosteroids depends on NMDARs

Activity‐dependent hippocampal long‐term synaptic plasticity in the CA1 area depends on NMDAR activation (Dudek and Bear 1992, 1993; Bear and Malenka 1994). To ascertain the involvement of these receptors in LTD and LTP induced by different stimulating protocols in the presence of AR and ER blockers, we performed electrophysiological recordings in the presence of AP‐5, the selective antagonist of NMDARs.

**Figure 5.**
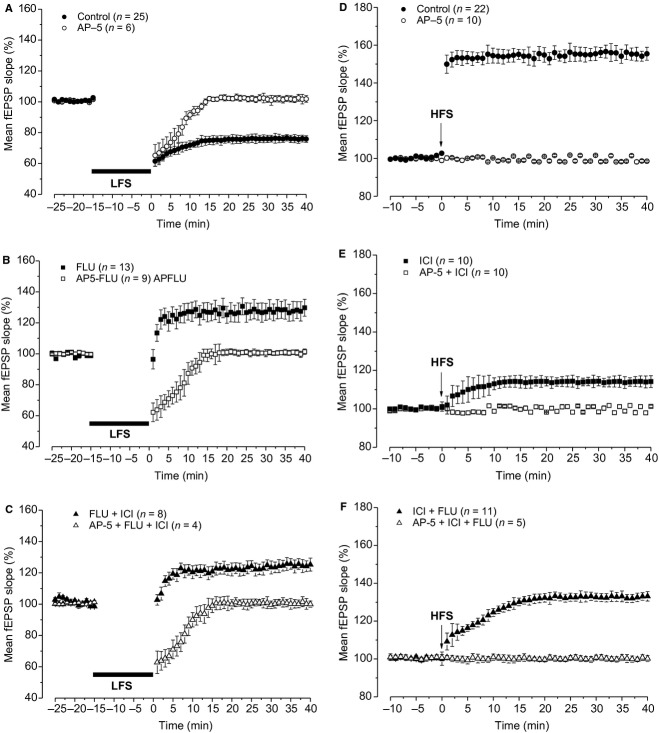
NMDAR dependence of synaptic plasticity under AR and ER blockade. Influence of AP‐5 on the effects of LFS (A–C) and HFS (D–F) in control condition and under AR and ER blockade (control: circles, flutamide: squares, flutamide plus ICI, triangles; filled symbols: without AP‐5, open symbols: with AP‐5). The bar and arrow show the LFS and HFS delivering time, respectively. AR, androgenic receptor; ER, estrogen receptor; LFS, low‐frequency stimulation; HFS, high‐frequency stimulation; ICI, ICI 182,780; *N*‐methyl‐d aspartate receptor.

As expected, AP‐5 prevented induction of both LTD and LTP in control condition (Fig. 5A and D). In fact, LFS delivered in the presence of AP‐5 in slices from young rats, produced a transient decrease in the fEPSP to 65.3 ± 6.2% (*n *=**6, three animals) that returned to the baseline (102.1 ± 2%) in few minutes (Fig. 5A), while in LTP recordings, AP‐5 completely prevented the HFS–LTP (100.1 ± 0.32%, *n *=**10, four animals, Fig. 5D).

Interestingly, AP‐5 also prevented the induction of LTP by LFS in the presence of either flutamide alone (*n *=**9, four animals) or flutamide plus ICI (*n *=**4, two animals). Like observed in the control condition, a transient depression of the fEPSP was only observed within the first 10–15 min after LFS application (66.5 ± 5.4% and 68.6 ± 6.4%, respectively) followed by a fEPSP recover to baseline values (100.5 ± 1.6% and 100.3 ± 2.6%, respectively, Fig. 5B and C).

Moreover, NMDAR blockade also prevented induction of the small LTP observed after HFS in the presence of ICI (99.9 ± 0.3, *n *=**10, five animals) and in the presence of ICI plus flutamide (100.7 ± 1.2, *n *=**5, three animals, Fig. 5E and F).

### PPF of the synaptic transmission is independent of AR or ER activation

**Figure 6.**
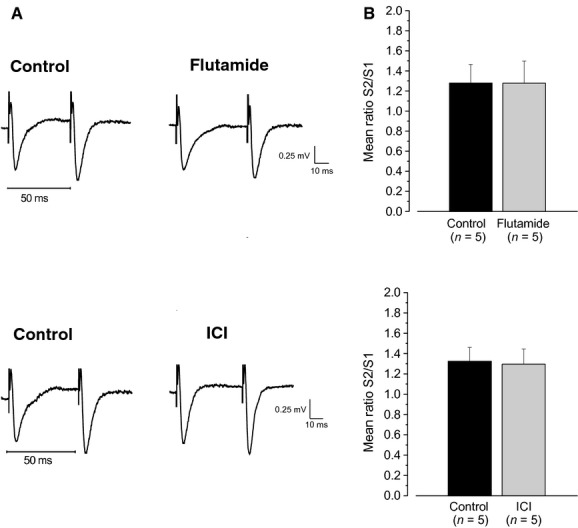
Influence of AR and ER blockade on the PPF. (A) Example traces showing fEPSPs recorded during a PPS inducing PPF (50 msec interval). (B) Histograms showing fEPSP ratio (mean ± SE,* n* = number of slices), in control condition (black columns) and 15 min after application of flutamide or ICI (gray column). ICI, ICI 182,780; PPF, paired‐pulse facilitation; fEPSP, field excitatory postsynaptic potential; PPS, paired‐pulse stimulation.

To gain insights on the synaptic mechanism controlling the effects of neurosteroids on ARs and ERs, we analyzed the PPF of synaptic transmission in control conditions (*n *=**5, three animals) and in the presence of flutamide (*n *=**5, three animals) or ICI (*n *=**5, three animals) at the same interstimulus interval (50 msec). Incubation of the slices with these drugs did not affect the PPR of fEPSP (control 1.27 ± 0.18 vs. flutamide 1.27 ± 0.22, Student's *t*‐test, *P* = 0.99; control 1.32 ± 0.13 vs. ICI 1.29 ± 0.14, Student's *t*‐test, *P *=**0.88, Fig. 6A and B), as well as the slope of the first and the second fEPSP (Student's *t*‐test, *P* > 0.05, not shown), suggesting the possible involvement of postsynaptic sites of action of neurosteroids in controlling long‐term changes of synaptic plasticity in the CA1 hippocampal area.

## Discussion

The results of this study support the hypothesis of an opposite role of estrogenic and androgenic neurosteroids in determining the sign of the activity‐dependent synaptic plasticity in the hippocampal CA1 neurons of male rats. In fact, we show for the first time that the induction of LTD by LFS is prevented by flutamide, the principally used antagonist for ARs, while LTP induced by HFS is markedly reduced by ICI, an antagonist of ERs. By contrast, flutamide and ICI had no effect on LTD and LTP once they are fully induced.

Regarding the induction of LTD, it was reverted into low‐amplitude LTP in the presence of flutamide, while the blockade of ERs with ICI had no effect. On the other hand, ICI impeded the full expression of LTP induced by HFS reducing its amplitude that, on the contrary, was not affected by flutamide. An interesting concern of this study is that the same results were obtained in both young (<P30) and adolescent (P50) rats, suggesting that, in spite of the age dependence of LTD and LTP development (Dudek and Bear 1993), the activation of ARs for LTD and ERs for LTP is anyhow involved.

These results suggest that androgenic and estrogenic neurosteroids are likely recruited during the specific synaptic activation inducing LTD or LTP, respectively, and facilitate these phenomena through interaction with their specific receptors.

The necessity of a selective activation of ERs by E2 for the full expression of hippocampal LTP seems to be confirmed by the fact that the synthesis of E2 is required for LTP, as shown by the same reduction in LTP caused by ICI and by the blocking agent for the P450‐aromatase (Grassi et al. 2011). In addition, the fact that LTP is not affected by flutamide excludes a role of ARs.

Similarly, we can exclude the role of ERs in the LTD induced by LFS, as ICI does not influence this phenomenon. Concerning the involvement of ARs in the induction of LTD, it is strongly supported by the annulment of LTD in the presence of flutamide. However, contrary to the involvement of estrogenic pathway in LTP, it seems difficult to prove the importance of androgenic pathway for LTD using enzymatic blockade. In fact, the 5*α*‐reductase antagonism can only prevent the synthesis of DHT from T, so that the accumulating T is able to act per se, by activating the ARs (Grassi et al. 2010a).

A helpful approach to assure the activation of androgenic signals in LTD and to exclude an interference of flutamide with other mechanisms could be to assay the effect of different anti‐ARs, but we presently know flutamide to be a very potent and broadly used AR antagonist.

Interestingly, the low‐amplitude LTP induced by HFS under ICI was slightly increased by flutamide, suggesting that HFS may not only activate potentiating estrogenic pathway but also the depressant androgenic pathway. Therefore, it is likely that when a full LTP is induced by HFS the estrogenic effect completely masks the depressant action of ARs, but when LTP is reduced, as occurs in the absence of ER activation, the androgenic inhibitory effect can be unmasked.

We found that the low‐amplitude LTP unveiled when LFS was applied under blockade of ARs, and the low‐amplitude LTP induced by HFS under blockade of ERs were both prevented by AP‐5, indicating their dependence on the NMDAR activation, as for LTP and LTD normally induced by HFS and LFS (Bear and Malenka 1994). On the basis of our findings, we can assert that activation of estrogenic or androgenic pathways, triggered by different neuronal activity, is necessary, in the CA1 hippocampal area of male rats, for determining the direction and the full expression of synaptic long‐term effects. In fact, the afferent stimulation, either at high or low frequency, in the presence of ER and AR blockade induced a smaller synaptic potentiation compared to LTP normally observed after HFS protocol. Therefore, we propose that the sign of an activity‐dependent synaptic modification can only be achieved in the presence of androgenic activation for LTD and estrogenic one for LTP. Therefore, it is likely that the determinant role of stimulation frequency in establishing the sign of synaptic long‐term effects depends on the specific synthesis of ARs by LFS and ERs by HFS.

Moreover, we found that ARs are also involved in DP, a long‐term LFS effect which can either reduce or cancel a previously induced LTP (Staubli and Lynch 1990; Dudek and Bear 1993). Although DP has been reported to occur more frequently in the adult than in the young animal (Dudek and Bear 1993), we observed it in both very young and adolescent rats and we found it to be fully prevented by flutamide, whereas ICI was ineffective.

How different stimulation frequencies can selectively activate the neural synthesis of ERs or ARs is a matter of future investigation, even though activation of specific enzymes mediating the androgenic or estrogenic conversion of T by different levels of Ca^2+^ at pre and/or postsynaptic sites has been suggested (Kimoto et al. 2001; Balthazart et al. 2006; Hojo et al. 2008). NMDAR‐mediated Ca^2+^ inflow into neurons following patterns of stimulation of afferents fibers at specific frequencies, is known to possibly drive LTP or LTD of synaptic plasticity in the hippocampus depending on the amount of the Ca^2+^ increase (Dudek and Bear 1992; Bear and Malenka 1994; Cummings et al. 1996). Accordingly, we suggest that in the absence of ER and AR activation, either LFS or HFS might induce LTP through a basal activation of NMDARs. We found that under blockade of ARs and ERs, LTP induced by LFS was smaller than the LTP induced by HFS. This could be due to a larger Ca^2+^ income associated to higher neuronal depolarization caused by HFS. Therefore, this basal LTP might be increased or reversed into LTD depending on the changes of Ca^2+^ levels leading to synthesis of ERs and ARs that might in turn produce a functional up‐ or downregulation of the NMDARs, respectively (Pouliot et al. 1996; Foy et al. 1999; Smith and McMahon 2005, 2006; Grassi et al. 2010b).

In addition, our findings suggest that the primary influence of androgenic and estrogenic signals is probably exerted at postsynaptic level, as blockade of either ARs or ERs did not affect the facilitated responses to paired stimuli, a short‐term and use‐dependent form of synaptic plasticity attributable to changes in neurotransmitter release probability (Manabe et al. 1993).

Ca^2+^ dynamics associated with intracellular enzymatic cascades that can be modulated by different types of afferent stimulation need to be explored in detail, as they might link the AR or ER activation with induction of NMDAR‐dependent LTD and LTP.

Moreover, interactions of estrogenic and androgenic neurosteroids with different molecular sites should also be taken into account and in particular with the GABA‐mediated transmission that is influenced by the sex neurosteroids (Murphy et al. 1998; Rudick and Wooley 2001; Reddy, 2004; Reddy and Jian 2010). In fact, it has been reported that E2 reduces GABAergic neurotransmission (Murphy et al. 1998; Rudick and Wooley 2001) while the downstream metabolites of DHT, like the androstane‐dioles, activate the GABA_A_ receptors (Reddy, 2004; Reddy and Jian 2010). Therefore, E2 might facilitate LTP through the contemporary increase in NMDAR response and decrease in GABAergic transmission, while DHT could also influence LTD by an increase in GABA_A_ mediated responses.

Despite the need for further mechanistic insight, this study demonstrates, for the first time, that estrogenic and androgenic neurosteroids play a crucial function in the induction and direction of the hippocampal synaptic plasticity, as ARs mediate the induction of LTD and DP by LFS and E2 of LTP by HFS. These effects were found to be age‐independent and more likely mediated by postsynaptic mechanisms involving NMDAR activation. However, we must consider that the effects observed in relatively young male rat may be influenced by different levels of circulating hormones depending on sex, estrous cycle and age, taking into account that the morphological and functional features of synaptic circuits are markedly governed by the history of estrogenic and androgenic impact on the neurons (Warren et al. 1995; Woolley et al. 1997; Good et al. 1999; McEwen 2002; Isgor and Sengelaub 2003; MacLusky et al. 2006). In addition, more recently, it has been shown that a long‐lasting block of locally produced E2, by prolonged administration of letrozole, slightly reduced LTP in male, while abolished LTP in female (Vierk et al. 2012). This effect of letrozole in male seems to be discordant with our results, as we show that LTP is remarkably reduced in male both in the presence of ER blockade and under letrozole, as reported in our previous study (Grassi et al. 2011). However, we observed the effect of acute blocks, while in the study by Vierk et al. (2012), the block was prolonged for hours and days preventing the action of E2 in maintaining the mature spine synapses. Therefore, the differences between the acute and chronic condition may suggest that, at least in male, E2 has a distinct impact on the mechanism inducing LTP and on that guiding synaptic formation. The study of Vierk et al. (2012) evidencing a different role of E2 in the maintenance of spine synapses in the hippocampus of female and male, suggests that we can expect sex differences in the rapid effect of local E2 on the LTP induction. Therefore, for a full description about the sex influence on the induction of LTP or LTD, we will investigate in the future the effect of blocking sex neurosteroid signals in female, also examining the possible modifications during the estrous cycle.

Moreover, it should be also worthy of not to analyze in future studies, the role of sex neurosteroids in LTP induced by different stimulation protocols, like the “pairing protocol” (Gustafsson et al. 1987; Chen et al. 1999).

Nevertheless, even if performed in a simplified experimental condition, this study gives clear evidence that the sign of glutamate synaptic plasticity can be determined by specific stimulation patterns in dependence on the local activation of estrogenic or androgenic pathways. In this context, neural T‐DHT and E2 seem to be very effective modulators of synaptic plasticity and might, therefore, significantly contribute to learning and memory processes.

## Acknowledgments

We thank E. Mezzasoma and M. Roscini for technical assistance.

## Conflict of Interest

None declared.
